# JMJD6 functions as an oncogene and is associated with poor prognosis in esophageal squamous cell carcinoma

**DOI:** 10.1186/s12885-023-11171-z

**Published:** 2023-07-24

**Authors:** Honggang Liu, Menglong Jiang, Fenghui Ma, Jiapei Qin, Xin Zhou, Liqun Xu, Xiaolong Yan, Tao Jiang

**Affiliations:** 1 Department of Thoracic Surgery, Tangdu Hospital, Air Force Medical University, 1 Xinsi Road, Xi’an, 710038 China; 2 Department of Thoracic Surgery, 1st Affiliated Hospital of Anhui Medical University, Hefei, 230022 China; 3 Medical Examination Center, Tangdu Hospital, Air Force Medical University, 1 Xinsi Road, Xi’an, 710038 China; 4 Department of Medical Oncology, Senior Department of Oncology, The Fifth Medical Center, Chinese PLA General Hospital, Beijing, China; 5 Department of Aerospace Medicine, Air Force Medical University, 169 Changle West Road, Xi’an, 710032 China

**Keywords:** Esophageal squamous cell carcinoma, Jumonji domain-containing protein 6, Immunohistochemical staining, Prognosis

## Abstract

**Background:**

Esophageal squamous cell carcinoma (ESCC) is one of the most common malignant tumors with a high prevalence and poor prognosis. It is an urgent problem to deeply understand the molecular mechanism of ESCC and develop effective diagnostic and prognostic methods.

**Methods:**

Using tumor tissue and corresponding paracancerous samples from 141 resected ESCC patients, we assessed Jumonji domain-containing protein 6 (JMJD6) expression using Immunohistochemical (IHC) staining. Kaplan-Meier survival analysis and univariate or multivariate analysis were used to investigate the relationship between JMJD6 expression and clinicopathological features. The expression status and prognostic value of JMJD6 were analyzed by bioinformatics and enrichment analysis.

**Results:**

The expression of JMJD6 in ESCC samples was higher than that in the corresponding paracancerous samples, and high expression of JMJD6 was positively associated with poor prognosis of ESCC patients. In addition, bioinformatics analysis of the expression and prognosis of JMJD6 in a variety of tumors showed that high expression of JMJD6 was significantly associated with poor overall survival (OS) in ESCC patients. Enrichment analysis indicated that the high expression of genes similar to JMJD6, such as *Conserved oligomeric Golgi 1(COG1)*, *Major facilitator superfamily domain 11* (*MFSD11*) and *Death Effector Domain Containing 2 (DEDD2)*, was associated with poor prognosis of ESCC, suggesting that JMJD6 might be involved in the occurrence and prognosis of ESCC.

**Conclusion:**

Our study found that JMJD6 expression was significantly increased in ESCC patients and positively correlated with prognosis, indicating that targeting JMJD6 might be an attractive prognostic biomarker and provides a potential treatment strategy for ESCC.

**Trial registration:**

The study was approved by Tangdu Hospital ethics committee (No. TDLL-202110-02).

**Supplementary Information:**

The online version contains supplementary material available at 10.1186/s12885-023-11171-z.

## Background

Esophageal cancer, the sixth leading cause of cancer-related death worldwide, is a complex disease whose etiology varies by histologic type and population [1]. Esophageal squamous cell carcinoma (ESCC) is the main histological subtype with an extremely high prevalence and poor prognosis in Asia, accounting for about 90% of the incidence of esophageal cancer [2]. In recent years, with the improvement of diagnosis and treatment of esophageal cancer, the 5-year survival rate of ESCC patients has been greatly improved [3]. However, the molecular mechanism by which JMJD6 promotes the progression of ESCC remains unclear and warrants further exploration to develop promising therapeutic approaches.

Jumonji domain-containing protein 6 (JMJD6), also known as phosphatidylserine receptor, is a member of Jumonji C domain-containing proteins [4]. JMJD6 has arginine demethylase and lysoyl hydroxylase activities in both histone and non-histone proteins [5, 6]. Furthermore, JMJD6 functions as a tyrosine kinase of histones, suggesting that JMJD6 acts at the transcriptional, splicing, posttranscriptional, and biochemical levels through chromatin configuration and epigenetic regulation [7]. Many studies have confirmed the role of JMJD6 in infection, inflammation, immunity, placental angiogenesis, tissue differentiation and embryonic development under normal physiological conditions [7, 8]. Abnormal expression of JMJD6 may contribute to the development of many diseases, such as neuropathic pain, foot-and-mouth disease, gestational diabetes, hepatitis C, and various types of cancer [7, 9–12]. Studies have shown that JMJD6, as an oncogene, is involved in the pathogenesis of many cancers, including oral cancer [13], colorectal cancer [14], breast cancer [6], lung cancer [15], and hepatocellular carcinoma [16] etc., and is closely related to the poor prognosis of patients. In addition, subsequent studies have demonstrated that JMJD6 has been implicated in the resistance of doxorubicin, methotrexate, etoposide and other chemotherapy drugs [7]. However, the role and molecular mechanism of JMJD6 in esophageal cancer, especially in ESCC, remain unclear. It would be important to continue efforts to elucidate the physiological functions of JMJD6 and the mechanisms by which JMJD6 contributes to ESCC progression.

To our knowledge, there has been no comprehensive analysis of the functional and clinical importance of JMJD6 in ESCC. In this study, we firstly systematically analyzed the expression status and prognostic value of JMJD6 by bioinformatics analysis in public datasets. Then we assessed JMJD6 expression and prognosis by immunohistochemical (IHC) staining of 141 paired ESCC samples and clinicopathological characteristics. Finally, Enrichment analyses found that JMJD6 may be involved in the occurrence and prognosis of ESCC. Our findings demonstrated that the expression of JMJD6 was significantly increased in ESCC patients and positively correlated with prognosis in ESCC. Given the important role of JMJD6 in cancer progression, we suggest that investigating JMJD6 may become an attractive diagnostic and prognosis biomarker and potential strategy to develop novel cancer therapeutics.

## Methods

### Database-mining

Data from the Gene Expression Profiling Interactive Analysis version 2 (GEPIA2) database (http://gepia2.cancer-pku.cn/) was analyzed, including JMJD6 expression, survival analysis, and correlation analysis. Furthermore, we used the Kaplan-Meier plotter database (https://kmplot.com/) to analyze the relationship between gene expression and prognosis of ESCC patients. Using STRING tool (version 11.5) (https://string-db.org/) to create a co-expression network of JMJD6.

### Gene expression analysis of JMJD6 and survival prognosis analysis

GEPIA2 is for analyzing RNA-sequencing data of tumor and normal tissue samples from the TCGA and Genotype-Tissue Expression projects using a standard processing pipeline [17]. A survival significance map of JMJD6 in various types of cancer, as well as overall survival (OS) Kaplan–Meier plots, was generated using the GEPIA2 “Survival Analysis” module. The expression threshold was 75% for high JMJD6 expression and 25% for low JMJD6 expression.

The Kaplan-Meier plotter can assess the impact of 54,000 genes on survival in a variety of cancer types. Gene expression and OS were downloaded from the Gene Expression Omnibus, European Genome-phenome Archive, and TCGA databases [18]. The purpose of this tool is to provide benefits in clinical decisions, healthcare policy and resource allocation based on a meta-analysis of biomarker assessments. The correlation between gene expression and survival in ESCC was analyzed using the Kaplan-Meier plotter. Hazard ratios (HR) with 95% confidence intervals and Logrank *p* values were also calculated.

### Patients and tissue samples

All ESCC cancer tissues and paired adjacent non-cancerous tissues were obtained from patients who underwent surgery of ESCC at Tangdu Hospital from January 2012 to June 2015. None of the patients received radiotherapy or chemotherapy before surgery, and the last follow-up was updated until death or June 2020. These ESCC cancer patients were diagnosed and graded on the basis of pathological features. The study was approved by the above-mentioned hospital ethics committee (No. TDLL-202110-02). This study was a retrospective study, and informed consent to participate was waived by Institution Review Board of Tangdu Hospital, Air Force Medical University.

### IHC

The 141 pairs of ESCC tissues and corresponding tumor-adjacent normal tissues were placed into a paraffin-embedded tissue microarray. The primary antibody of anti-JMJD6 (1:1000, GB11341, Servicebio) was used for IHC staining. We applied the colon tissue section known to express JMJD6 as a positive control, and our IHC staining result was consistent with that of the Human Protein Atlas. As a negative control, we used sections of thymus tissue that were negative for JMJD6 expression. The positive and negative controls that have been run simultaneously with our study samples during IHC staining, which are shown in Fig. S3a. The IHC intensity scores included negative (score 0), weak (score 1), moderate (score 2), or strong staining (score 3). The proportion of positive stains was scored as 0 (< 5%), 1 (6–25%), 2 (26–50%), 3 (51–75%), or 4 (> 75%). The diagram of each staining pattern in IHC intensity scores (0/1/2/3) in tumor-adjacent normal/cancer tissue is shown in Fig. S3b. Multiply the two scores to get the total score. IHC staining was read by expert pathologists, and all pathologists were blinded to clinical data prior to statistical analysis. ESCC samples were divided into low or high JMJD6 expression groups according to their respective mean scores.

### JMJD6-Related gene enrichment analysis

The STRING tool was used to create a co-expression network of Homo sapiens JMJD6. The main parameters were as follows: Active interaction source: network; meaning of network edges: evidence; maximum number of interactors: 100; and minimum required interaction score: high confidence (0.700). The platform generated graphs showing the top 14 proteins significantly co-expressed with JMJD6 expression in different types of cancer. The GEPIA2 “Similar Gene Detection” module was used to extract the 100 genes with the most similar expression patterns to JMJD6. Using the gene symbols of these 100 genes as input gene symbols in OmicShare, gene ontology pathway enrichment analysis was performed for biological processes, cellular components, and molecular functional categories. In addition, gene correlation analysis was performed using the GEPIA2 “Correlation Analysis” module.

### Statistical analysis

SPSS 23.0 software was used for statistical analysis. The relationship between JMJD6 expression and clinicopathological features in patients with ESCC was evaluated by the χ2 test or Fisher’s exact test. Survival analyses were assessed using the Kaplan-Meier method and Log-rank test. Cox proportional hazards model was used for univariate and multivariate survival analysis. The student’s t-test was used for comparison between two groups. Data was presented as mean ± standard deviation (SD). A value of *p* < 0.05 was considered as statistically significant difference.

## Results

### Correlation of JMJD6 expression and ESCC prognosis and survival

**Fig. 1:**
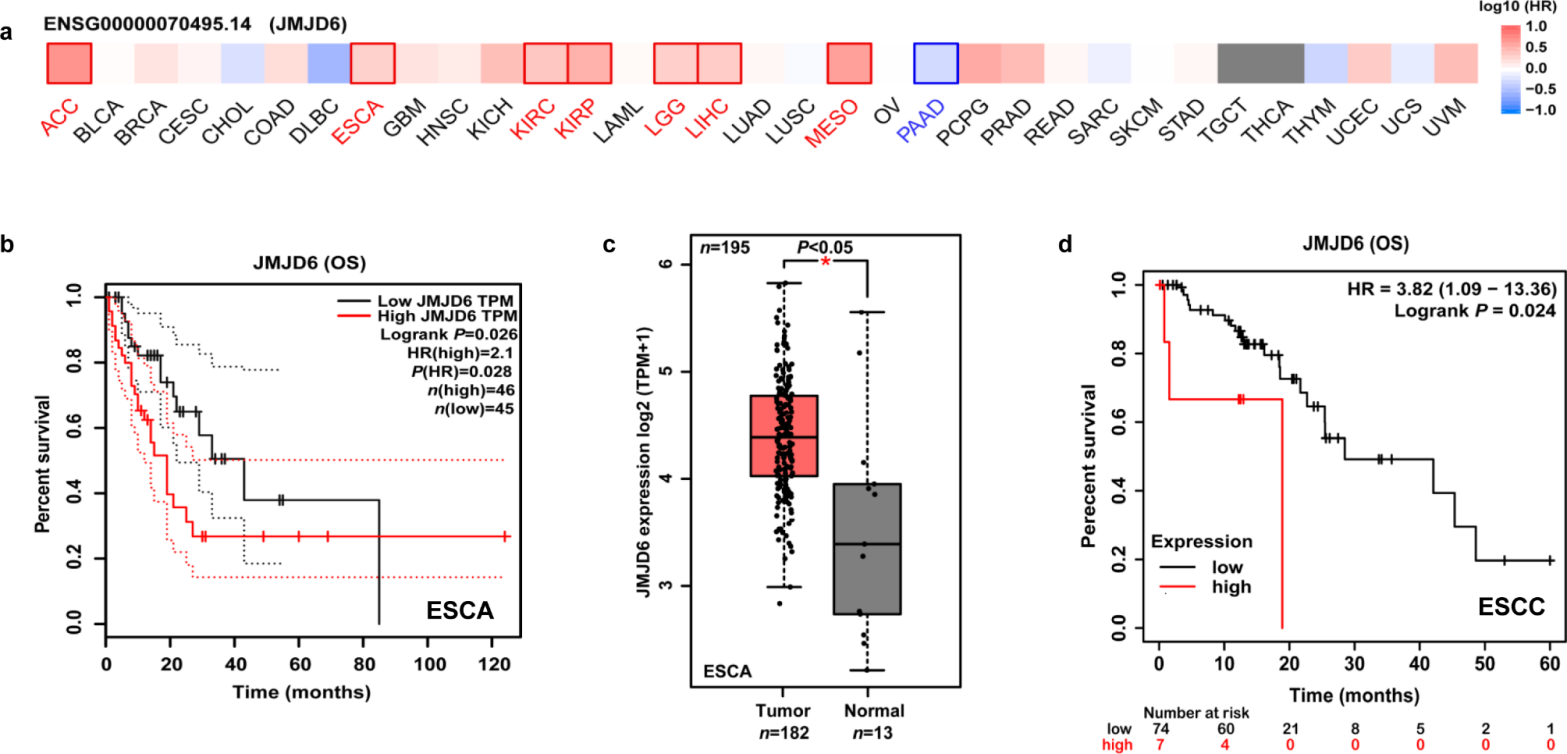
Correlation between JMJD6 expression and OS in patients with different tumor types. GEPIA2 was used to construct (**a**) a survival map and perform (**b**) OS analysis, as well as (**c**) a statistical plot of JMJD6 expression in ESCA and non-tumor tissues. (**d**) Kaplan-Meier plotter analysis showed that ESCC patients in the JMJD6-high group had shorter OS than those in the JMJD6-low group. The survival map and Kaplan–Meier plots with significant results are shown. The 95% confidence intervals of overall survival are indicated by red and black dotted lines for high and low JMJD6 groups, respectively. Abbreviations: ESCA, esophageal carcinoma; ESCC, Esophageal squamous cell carcinoma; GEPIA2, Gene Expression Profiling Interactive Analysis version 2; JMJD6, Jumonji domain-containing protein 6; OS, overall survival

To assess the association between JMJD6 and prognosis, we investigated human tumors using GEPIA2. The relationship between JMJD6 and prognosis varied across different types of cancers. In most cancers, higher JMJD6 expression was associated with shorter OS in cases of adrenocortical carcinoma (ACC) (HR = 5.3, Logrank *p* = 0.017), esophageal carcinoma (ESCA) (HR = 2.1, Logrank *p* = 0.026), kidney renal clear cell carcinoma (KIRC) (HR = 2.4, Logrank *p* = 0.00015), kidney renal papillary cell carcinoma (KIRP) (HR = 3.3, Logrank *p* = 0.014), brain lower grade glioma (LGG) (HR = 2.1, Logrank *p* = 0.0033), liver hepatocellular carcinoma (LIHC) (HR = 2.2, Logrank *p* = 0.00079), mesothelioma (MESO) (HR = 4.4, Logrank *p* = 5.9e-05) (Fig. 1a and b and Fig. S1). In addition, based on datasets of GEPIA2, we found that JMJD6 was highly expressed in ESCA compared with non-tumor tissues (*p* < 0.05) (Fig. 1c). As ESCC is the main histological subtype of ESCA and the prevalence is extremely high in Asia, especially in China, we further used Kaplan-Meier plotter to analyze survival for confirmation of the association between JMJD6 and prognosis of ESCC. Patients in the JMJD6-high group had shorter OS than those in the JMJD6-low group with ESCC (HR = 3.82 [1.09–13.36], Logrank *p* = 0.024) (Fig. 1d). These findings indicated that JMJD6 expression was elevated in ESCC and it most likely served as a biomarker for poor prognosis.

To verify the role of JMJD6 in ESCC, we analyzed whether its expression was correlated with clinicopathological variables. We performed Cox proportional hazard regression analysis of the patients’ OS. Univariate analysis showed that JMJD6 expression (HR = 2.282 [1.509–3.451], *p <* 0.001), lymphatic metastasis (HR = 1.806 [1.204–2.710], *p =* 0.004), and tumor-node-metastasis (TNM) stage (HR = 1.731 [1.150–2.605], *p =* 0.008) were associated with ESCC patients’ survival (Table 1). Furthermore, multivariate Cox regression analyses showed that JMJD6 was an independent prognostic factor for ESCC patients (HR = 2.113 [1.388–3.218], *p <* 0.001, Table 1). These results demonstrated that higher JMJD6 expression was associated with poor prognosis in patients with ESCC and the promotion of lymph node metastasis might be an important factor.

**Table 1:** Univariate and multivariate analyses of prognostic factors for ESCC patient survival

	Univariate analysis			Multivariate analysis		
Variables	HR	95% CI	*P* value	HR	95% CI	*P* value
Age (≥ 60 years/<60 years)	1.265	0.814–1.965	0.296			
Gender (Male/Female)	1.051	0.622–1.775	0.853			
Differentiation (Poor/Well and moderate)	1.027	0.560–1.884	0.930			
Lymphatic metastasis (N1-N3/N0)	1.806	1.204–2.710	0.004	1.412	0.507–3.934	0.509
pTNM stage (III/I-II)	1.731	1.150–2.605	0.008	1.140	0.407–3.189	0.803
JMJD6 expression (High/Low)	2.282	1.509–3.451	< 0.001	2.113	1.388–3.218	< 0.001

### Expression of JMJD6 in ESCC patients

**Fig. 2:**
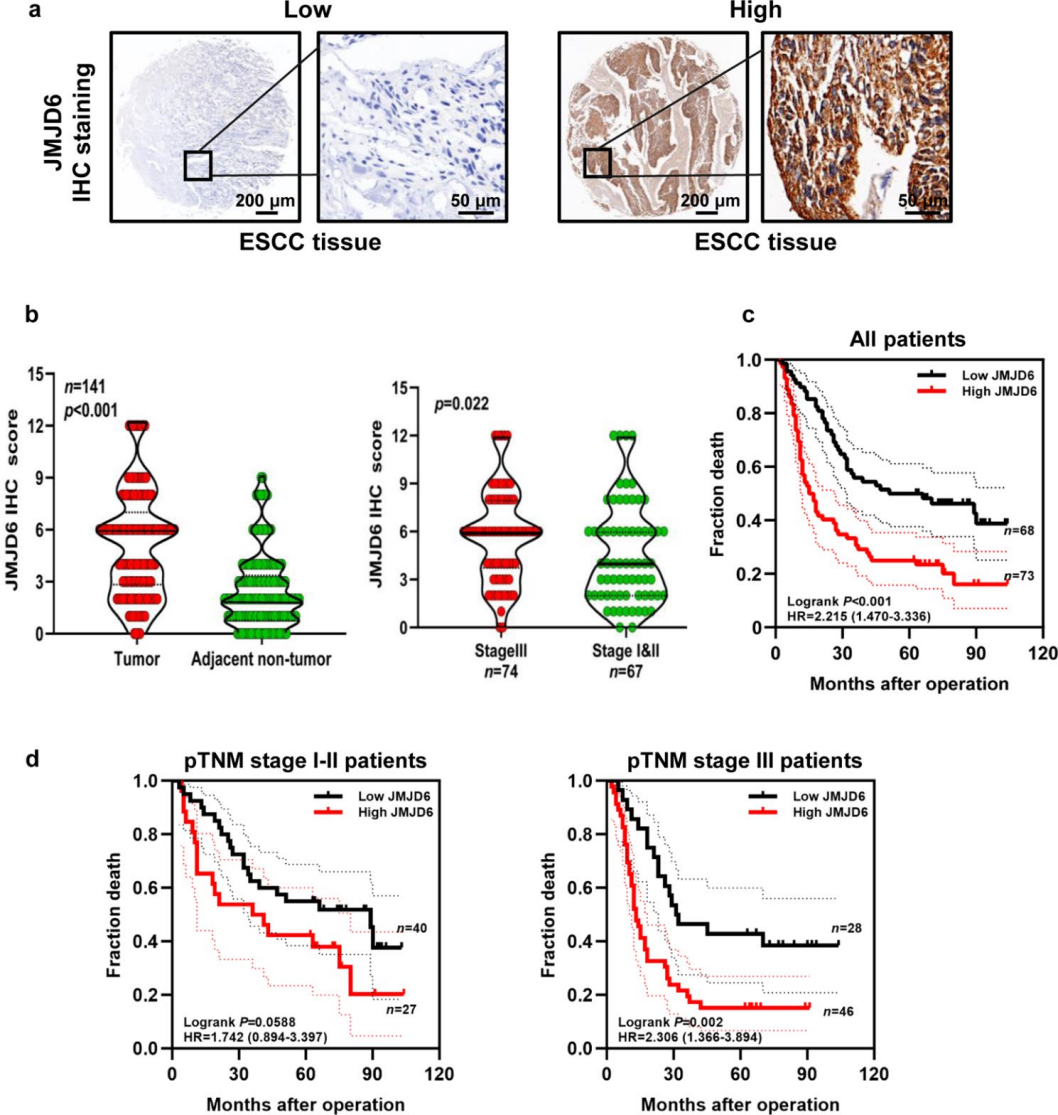
High JMJD6 expression in ESCC tissues is associated with poor prognosis. (**a**) Representative IHC image of JMJD6 expression in ESCC. Scale bars, 200 and 50 μm (inset). (**b**) The expression of JMJD6 in 141 patients with ESCC was statistically analyzed by IHC staining results based on tissue microarray. (**c**-**d**) Kaplan-Meier survival analyses of high or low JMJD6 expression based on tissue microarray IHC results for 141 patients (**c**), 67 ESCC patients in the early clinical stages, and 74 ESCC patients in the late clinical stage (**d**). Abbreviations: ESCC, Esophageal squamous cell carcinoma; IHC, Immunohistochemical; JMJD6, Jumonji domain-containing protein 6

To further validate the JMJD6 protein level in ESCC tissues, we detected JMJD6 expression using IHC analysis in a tissue microarray containing 141 paired ESCC tumor-normal tissues (Fig. 2a). As shown in Fig. S3, sections of colon tissue that is known to express JMJD6 were used as a positive control, and sections of thymic tissue that is negative for JMJD6 were used as a negative control, and each staining pattern in IHC intensity score (0/1/2/3) of tumor-adjacent normal/cancer tissue is shown. The expression of JMJD6 in ESCC tissues was significantly higher than that in adjacent noncancerous samples (*p <* 0.05, Fig. 2b). In addition, ESCC patients with lymph node metastasis (N1-3), poorly differentiated, and a high American Joint Committee on Cancer (AJCC) 7th stage (stage III) had a significantly higher JMJD6 expression than those without lymph node metastasis (N0), with well and moderate differentiation and a lower 7th AJCC stage (stage I/II) (*p* < 0.05, Table 2). Kaplan–Meier analysis also showed that ESCC patients with high JMJD6 expression had a shorter OS (HR = 2.215 [1.470–3.336], Logrank *p* < 0.001) (Fig. 2c). Furthermore, our results also indicated that the prognosis of ESCC patients with high JMJD6 expression was poor in the late clinical stage (stage III) (HR = 2.306 [1.366–3.894], Logrank *p* = 0.002). However, there was no significant difference in prognosis in the early clinical stage (stage I/II) (HR = 1.742 [0.894–3.397], Logrank *p* = 0.0588) (Fig. 2d).

**Table 2:** Association of JMJD6 expression with clinicopathological parameters of patients with ESCC

	JMJD6 expression			
**Category**	***n***	**low**	**high**	***p*** **value**
Age				0.777
< 60	44	22	22	
>=60	97	46	51	
Gender				0.841
Male	115	55	60	
Female	26	13	13	
Tumor invasion				0.464
T1-T2	12	7	5	
T3-T4	129	61	68	
Lymphatic metastasis				0.005
N0	74	44	30	
N1-N3	67	24	43	
Differentiation				0.040
Well and moderate	122	63	59	
Poor	19	5	14	
pTNM stage				0.009
I-II	67	40	27	
III	74	28	46	

### JMJD6-Related gene enrichment analysis

**Fig. 3:**
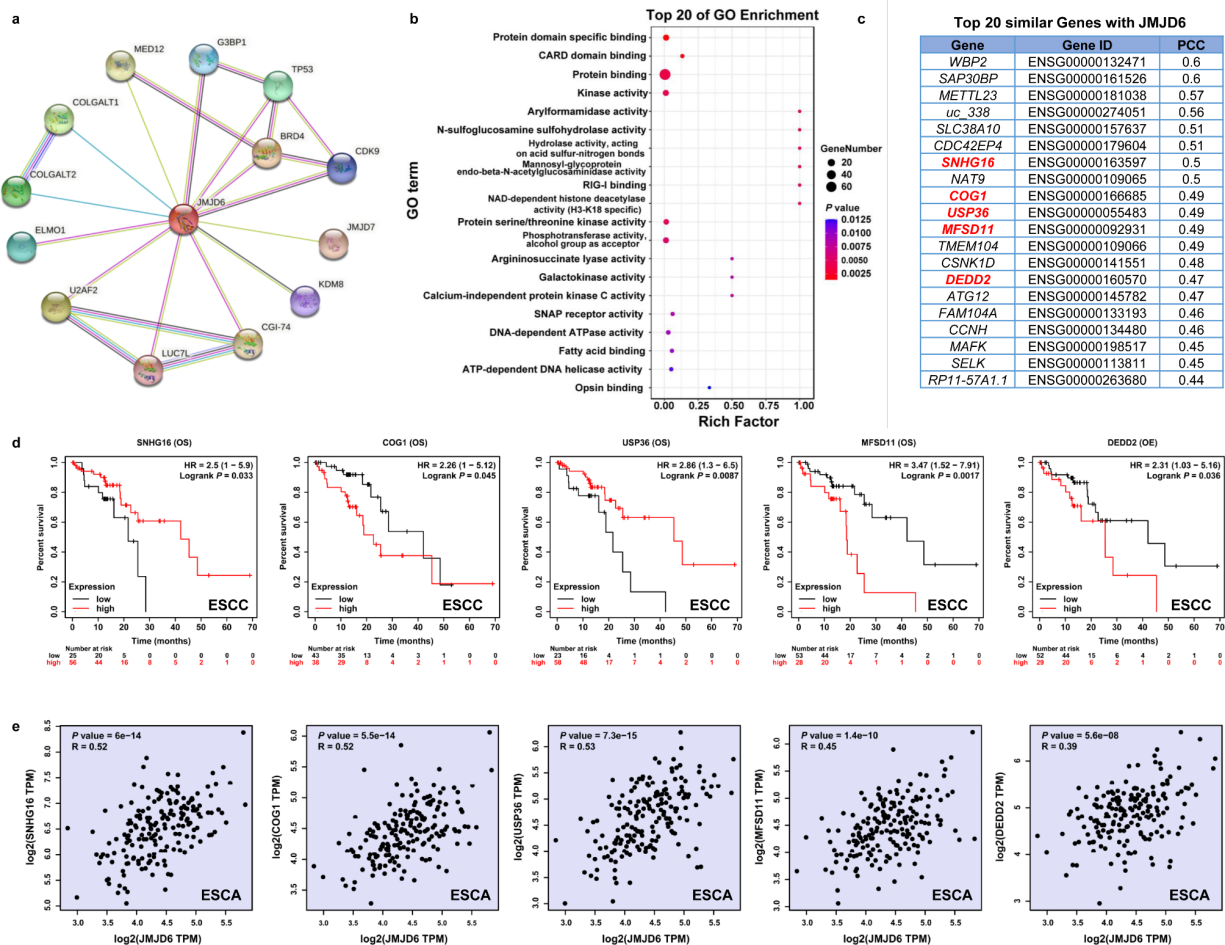
JMJD6-related gene enrichment analysis. (**a**) Co-expression network of 14 proteins co-expressed with JMJD6 obtained by the STRING tool. (**b**) Gene Ontology analysis of the genes similar to *JMJD6* obtained by the GEPIA2. (**c**) The top 20 genes similar to *JMJD6* obtained by the GEPIA2. (**d**) Kaplan-Meier survival analysis of *COG1*, *MFSD11*, *DEDD2*, *SNHG16* and *USP36* expression from the Kaplan–Meier plotter database. (**e**) Correlation analysis between *JMJD6* and *COG1*, *MFSD11*, *DEDD2*, *SNHG16* and *USP36* conducted by GEPIA2 across ESCA. Abbreviations: *COG1, Conserved oligomeric Golgi 1*; *DEDD2, Death Effector Domain Containing 2*; ESCA, esophageal carcinoma; ESCC, Esophageal squamous cell carcinoma; JMJD6, Jumonji domain-containing protein 6; *MFSD11, Major facilitator superfamily domain 11*; *SNHG16, Small nucleolar RNA host gene 16*

To investigate the functional mechanism of JMJD6 in carcinogenesis, we used STRING tool to extract the top 14 proteins co-expressed with JMJD6. JMJD6 physically interacts with bromodomain-containing protein 4 (BRD4), mediator of RNA polymerase II transcription subunit 12 homolog (MED12), cyclin-dependent kinase 9 (CDK9), lysine demethylase 8 (KDM8), TP53 and etc. (Fig. 3a), which have well-characterized functions in tumorigenesis [19–23]. For example, BRD4 plays an important role in super-enhancer organization and regulation of oncogene expression. Furthermore, MED12 and BRD4 could cooperate to sustain cancer growth upon loss of mediator kinase [24]. Following that, we used GEPIA2 “Similar Gene Detection” module to extract the top 100 genes with expression patterns similar to *JMJD6* from ESCA in the TCGA datasets. Gene Ontology enrichment analysis indicated that these genes were closely linked to protein binding and protein serine/threonine kinase activity (Fig. 3b). The top 20 genes with the most similar expression patterns to JMJD6 in ESCA were shown and the prognostic value of these genes was explored in the website of Kaplan-Meier plotter (Fig. 3c and Fig. S4). A high expression of *Conserved oligomeric Golgi 1(COG1)* (HR = 2.26 [1-5.12], Logrank *p* = 0.045), *Major facilitator superfamily domain 11* (*MFSD11*) (HR = 3.47 [1.52–7.91], Logrank *p* = 0.0017), *Death Effector Domain Containing 2 (DEDD2)* (HR = 2.31 [1.03–5.16], Logrank *p* = 0.036) while a low expression of *Small nucleolar RNA host gene 16 (SNHG16*) (HR = 2.5 [1-5.9], Logrank *p* = 0.033), *USP36* (HR = 2.86 [1.3–6.5], Logrank *p* = 0.0087) was related to a worse OS in ESCC patients (Fig. 3d). However, according to the “Correlation Analysis” module of GEPIA2, we found that the expression of JMJD6 was strongly positively correlated with the expression levels of *COG1*, *MFSD11*, *DEDD2*, *SNHG16 and USP36* in ESCA (Fig. 3e). Based on these results, we speculated that JMJD6 might play a tumor-promoting role in ESCC.

## Discussion

Despite improvements in treatment efficacy over the last 10 years, the diagnostic efficiency and the prognosis of patients with ESCC are still poor due to limited biomarkers and tumor aggressiveness. In order to find new biomarkers for early diagnosis and prognosis, we comprehensively analyzed the clinical value and prognostic role of JMJD6 in a variety of cancers, especially in ESCC by mining the database. Our results demonstrated that the expression of JMJD6 was significantly increased compared with normal tissue, and was positively correlated with lymph node metastasis and TNM stage. In addition, we used bioinformatics analysis to find that ESCC patients with high JMJD6 expression had a shorter OS. However, both GEPIA2 database and Kaplan-Meier plotter database showed no correlation between JMJD6 expression and RFS in ESCC patients (Fig S2). We further reviewed the literature and found high JMJD6 expression was associated with improved OS and RFS in cholangiocarcinoma, which indicated that JMJD6 was one of the improved independent prognostic factors of OS and RFS [25].

JMJD6 is an Fe(II)- and2-oxoglutarate-dependent oxygenase and plays a crucial role in the differentiation of various tissues and cells during embryogenesis, and it knockout exhibited abnormal neurodevelopmental phenotypes in mice [16]. Although the exact mechanism by which JMJD6 promotes tumorigenesis and progression has not been elucidated, the interaction of JMJD6 with cancer-related signaling pathways has been identified as one of the potential mechanisms. Previously study have shown that JMJD6 expression was higher in human oral squamous cell carcinoma by inducing interleukin 4 transcription and binding to its promoter region [13]. Inhibition of JMJD6 could restore hepatocyte nuclear factor 4 alpha levels in protein arginine methyl transferase 1-knockout hepatocytes and prevent increased hepatocyte proliferation [26]. Furthermore, JMJD6 could form protein complexes with N-Myc and BRD4 and regulate the transcription of E2F2, N-Myc and c-Myc. Knockdown of JMJD6 inhibited the proliferation and survival of neuroblastoma cells and tumor progression in mice [27]. Furthermore, high-level expression of JMJD6 in human neuroblastoma and lung adenocarcinoma (EAC) independently predicted poor patient prognosis [27, 28]. Our findings indicated that the expression of JMJD6 was increased in ESCC and higher JMJD6 expression was associated with poor prognosis. Furthermore, ESCC patients with high JMJD6 expression have a poor prognosis in the late clinical stage, but there is no significant difference in the early clinical prognosis. A recent study found that the expression of JMJD6 in prostate cancer was up-regulated with the progression of stage and grade [29]. High expression of JMJD6 was closely related to advanced clinicopathologic stage, strong invasiveness and poor prognosis of melanoma [30]. JMJD6 overexpression could induces EMT, and greatly enhances tumor metastasis in neuroblastoma and breast cancer [9, 27]. These results suggest that JMJD6 is involved in promoting cell transformation, tumor progression, and metastasis. In addition, considering the potential value of JMJD6 in cancer therapy, an inhibitor SKLB325 has been designed based on the crystal structure of the JmjC domain of JMJD6, which has shown significant anti-tumor effects in ovarian cancer [31]. It could be speculated that targeting JMJD6 might be a potential strategy for developing new therapies for ESCC.

Gene enrichment analysis showed that proteins co-expressed with JMJD6 were strongly associated with tumor progression, such as BRD4, MED12, CDK9, KDM8, TP53 and etc. Kaplan-Meier Plotter website was used to explore the prognostic value of the top 20 genes similar to JMJD6, and 5 genes were found to be statistically significant, all of which were positively correlated with JMJD6 expression in ESCA. However, high expression of *COG1*, *MFSD11*, *DEDD2* while low expression of *SNHG16* and *USP36* was related to the poor prognosis of ESCC. We speculated that this result might be related to the fact that ESCC and EAC are two subtypes of esophageal cancer with different epidemiology and pathogenesis, different molecular profiles, as well as different therapeutic targets. In addition, we found that the genes similar to JMJD6 might act as oncogenes in the carcinogenic progression and are highly correlated with poor prognosis in multiple cancers.

COG1, a heteromeric subunit of the COG complex, plays a crucial role in endosomal to Golgi transport, retrograde transport of Golgi vesicles, and Golgi homeostasis [32]. Various studies have demonstrated that COG complex plays an important role in tumor metastasis by regulating protein glycosylation and is associated with tumor grading and prognosis [33, 34]. Furthermore, COG complex dysfunction affects the dissociation of glycosyltransferases from anterograde cargo molecules and interferes with normal protein glycosylation [35]. Although the role of COG complex in tumor has not been reported, abnormal expression of COG complex can affect tumor invasion and metastasis by regulating protein glycosylation [32]. *MFSD11* gene encoded protein contained major facilitator superfamily domain, this superfamily was a diverse group of secondary transporters [36]. However, the Uniprot website states that MFSD11 is related to the unc-93 family and not to the major promoter superfamily. There are less studies on MFSD11, thus the mechanism and role of MFSD11 are still unclear. A spearman correlation analysis showed that MFSD11 was most significantly related to survival in ovarian cancer [37].

DEDD2, is a DEDD homolog, including an NH2-terminal region with a DED domain, and a COOH-terminal region with some DNA-binding proteins [38]. DEDD2 is involved in regulating the degradation of intermediate filaments during apoptosis [39]. In addition, along with DEDD, DEDD2 is a strong inducer of death receptor-induced apoptosis [38]. It has been involved in a variety of malignancies. Studies have found that DEDD2 could downregulate miR-301 to help luteolin inhibit prostate cancer cell proliferation and induce apoptosis [40]. Furthermore, DEDD2 contributes to apoptosis of non-small cell lung cancer and triple-negative breast cancer cells [41]. Bioinformatics analysis found that the expression of *COG1*, *MFSD11* and *DEDD2* was positively correlated with JMJD6 and the poor prognosis of ESCC, which further proved the role of JMJD6 in the biological behavior of ESCC and deserved further exploration.

## Conclusions

In a nutshell, we aimed to investigate JMJD6 in the occurrence and prognosis of ESCC through clinical data and bioinformatics analysis in public datasets. The obtained data indicated that high JMJD6 expression was significantly related with poor OS in ESCC patients. In addition, the analysis between JMJD6 expression and clinicopathological features as well as IHC staining of ESCC patients indicated that JMJD6 was a promising prognostic biomarker for ESCC patients. However, the mechanism of JMJD6 as a tumor-associated protein in ESCC is not sufficient, which need more researches to be further elucidated.

## Electronic supplementary material

Below is the link to the electronic supplementary material.

Supplementary Material 1

## Data Availability

The dataset generated or analyzed during the current study are available in the GEPIA2 (http://gepia2.cancer-pku.cn/), Kaplan-Meier plotter database (https://kmplot.com/), and STRING tool (https://string-db.org/). The other data used during the current study are available from the corresponding author.

## List of abbreviations

COG1: Conserved oligomeric Golgi 1
DEDD2: Death Effector Domain Containing 2
ESCA: Esophageal carcinoma
ESCC: Esophageal squamous cell carcinoma
GEPIA2: Gene Expression Profiling Interactive Analysis version 2
IHC: Immunohistochemical
JMJD6: Jumonji domain-containing protein 6
MFSD11: Major facilitator superfamily domain 11
OS: Overall survival
SNHG16: Small nucleolar RNA host gene 16
