# Supplementary material for: JMJD6 functions as an oncogene and is associated with poor prognosis in esophageal squamous cell carcinoma
Source: BMC Cancer. 2023 Jul 24;23:696. doi: 10.1186/s12885-023-11171-z (PMC10367331; doi:10.1186/s12885-023-11171-z)
Supplement: Supplementary file 1 — Supplementary Material 1 [file 12885_2023_11171_MOESM1_ESM.docx]

**Supplementary Materials for**

**JMJD6 Functions as an Oncogene and is Associated with Poor Prognosis in Esophageal Squamous Cell Carcinoma.**

Honggang Liu^1*^, Menglong Jiang^2*^, Fenghui Ma^3*^, Jiapei Qin^4^, Xin Zhou^4^, Liqun Xu^4#^, Xiaolong Yan ^1#^, and Tao Jiang^1#^

^#^To whom correspondence should be addressed: yanxiaolong@fmmu.edu.cn (Xiaolong Yan), aliqunxu@fmmu.edu.cn (Liqun Xu), and jiangtaochest@163.com (Jiang Tao).

Figure S1：


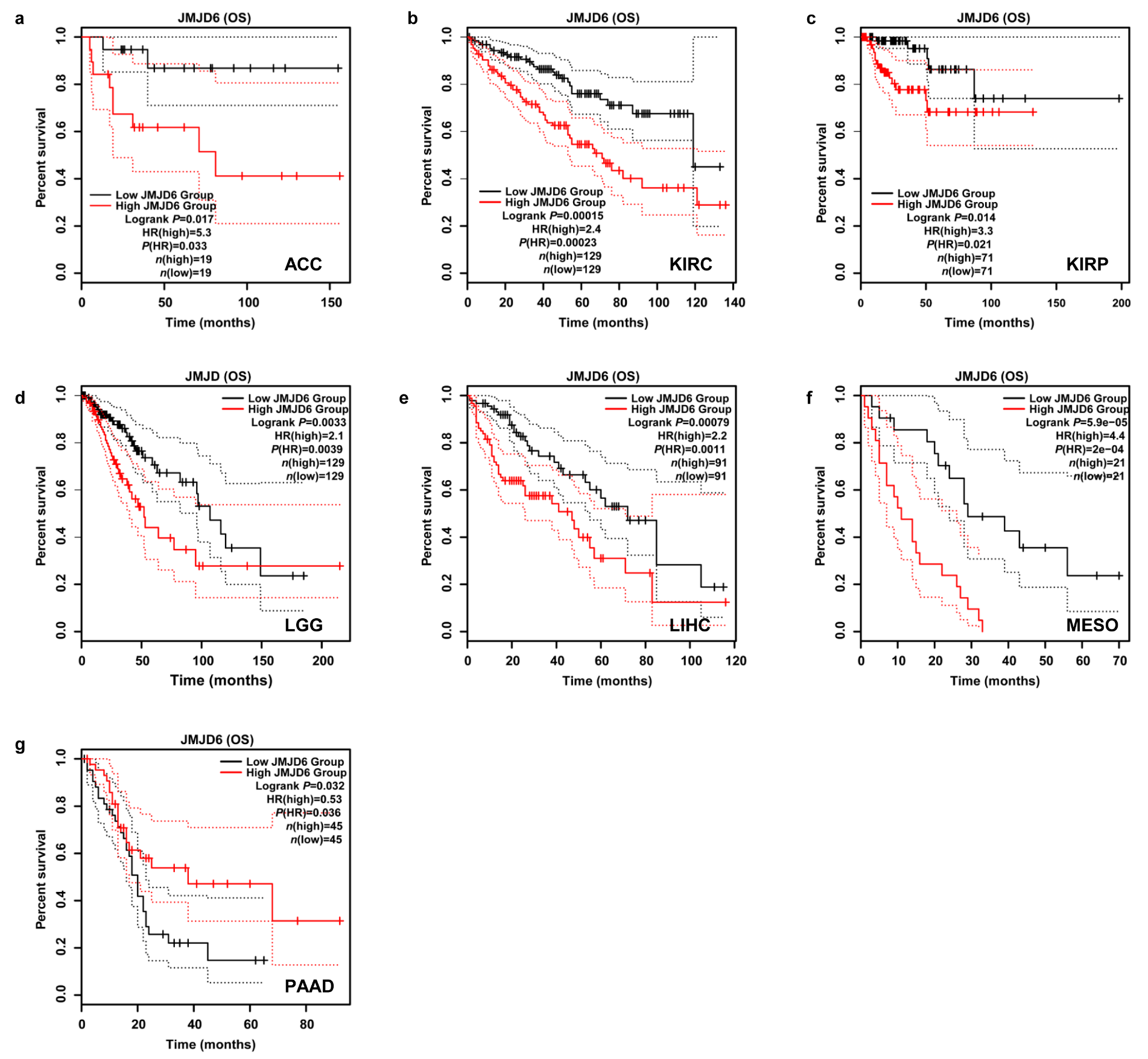


**Fig.S1.** Correlation between JMJD6 expression and OS in patients with different tumor types. (a-g) GEPIA2 was used to perform OS analysis. Abbreviations：GEPIA2, Gene Expression Profiling Interactive Analysis version 2; JMJD6, Jumonji domain-containing protein 6; OS, overall survival.

Figure S2：


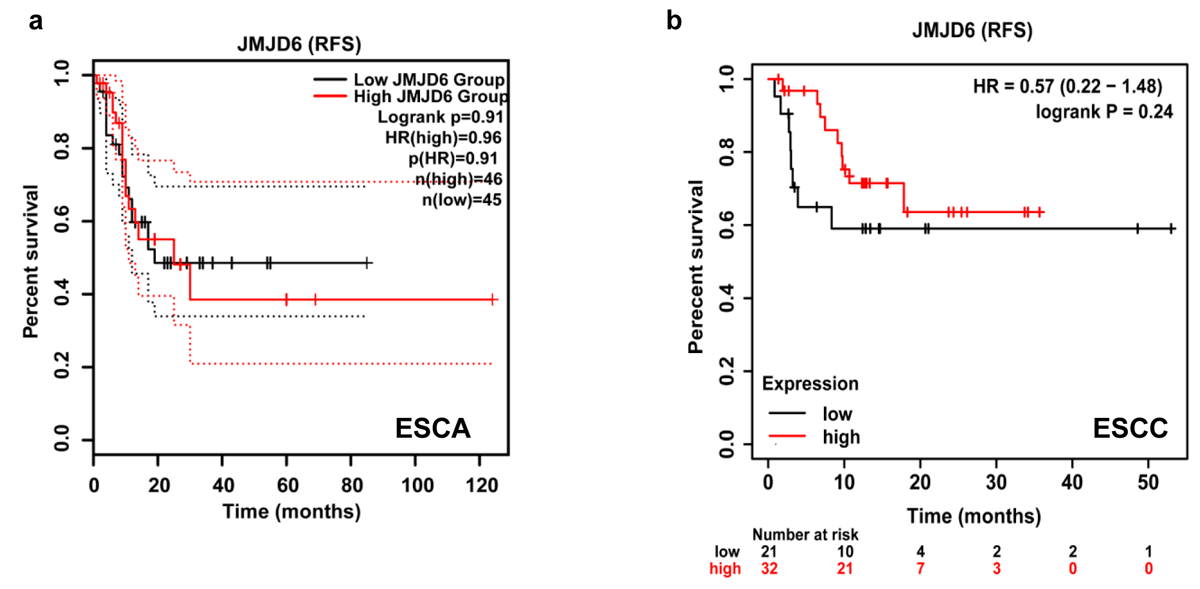


**Fig.S2.** Correlation between JMJD6 expression and RFS in ESCC patients. (a) GEPIA2 was used to perform RFS analysis. (b) Kaplan-Meier plotter analysis of high or low JMJD6 expression in ESCC patients based on Kaplan-Meier plotter. Abbreviations：ESCC, Esophageal squamous cell carcinoma; GEPIA2, Gene Expression Profiling Interactive Analysis version 2; JMJD6, Jumonji domain-containing protein 6; RFS, recurrence-free survival

Figure S3：


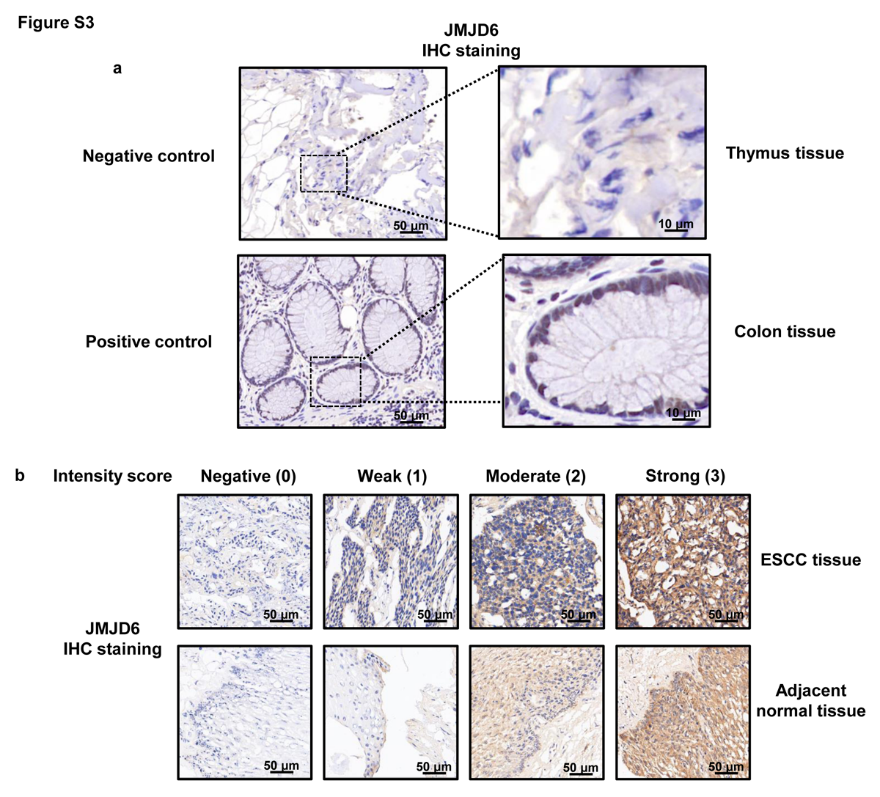


**Fig.S3.** The diagram of each staining pattern in IHC. (a) The negative and positive controls for IHC staining. Scale bars, 50 μm and 10 μm (inset). (b) The intensity scores in tumor-adjacent normal/cancer tissue used for IHC. The IHC intensity scores include 0 (negative), 1 (weak), 2 (moderate), or 3 (strong). Scale bars, 50 μm (inset). Abbreviations：ESCC, Esophageal squamous cell carcinoma; IHC, Immunohistochemical; JMJD6, Jumonji domain-containing protein 6.

Figure S4：


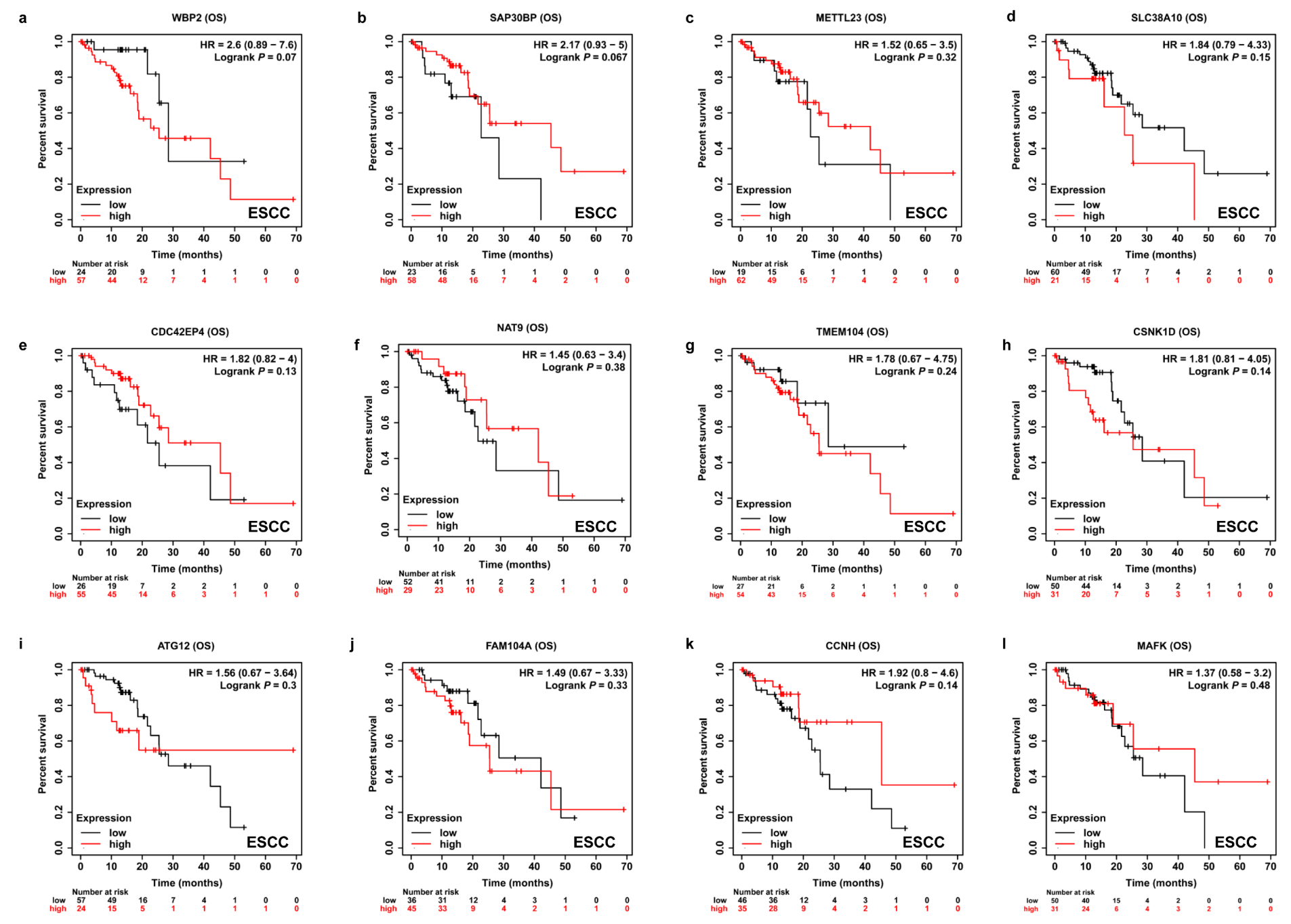


**Fig.S4.** Kaplan-Meier survival analysis of genes expression from the Kaplan-Meier plotter database. Abbreviations：ESCC, Esophageal squamous cell carcinoma.
